# Influence of pine and miscanthus biochar on the water activity and moisture sorption isotherms of used broiler litter

**DOI:** 10.1016/j.heliyon.2023.e22618

**Published:** 2023-11-20

**Authors:** John Emerson Linhoss, Maryam Kate Mohammadi-Aragh, Jeffrey Dwayne Evans

**Affiliations:** a *Biosystems Engineering, Auburn University, 350 Mell Street, Auburn, AL, 36849, USA; b Agricultural and Biological Engineering, Mississippi State University, 130 Creelman St., Mississippi State, MS, 39762, USA; c USDA-ARS Poultry Research Unit, 606 Spring St., Starkville, MS, 39759, USA

**Keywords:** Poultry, Biochar, Water activity, Litter management, Isotherm

## Abstract

The effect of various inclusion rates of pine biochar (PBC), miscanthus biochar (MCB) and starting moisture contents on the water activity (A_w_) of broiler litter/biochar mixtures was examined. Suitable models for the resulting moisture sorption isotherms were also evaluated. For poultry mixed with PBC and MBC, higher starting moisture contents led to increased A_w_. A_w_ generally increased at high inclusion rates of PBC and MBC, which may be explained by the porous nature and general hydrophobicity of BC. Of the five models examined, the exponential rise to maximum was considered to be the most useful due to its accuracy at moisture contents similar to those commonly found in commercial poultry houses (10–30 % wet basis). Accuracy of models was determined using R^2^, mean squared prediction error (MSPE), and mean absolute percent error (MAPE). In general, higher inclusion rates of biochar resulted in less overall prediction accuracy. Results from this paper indicate that several moisture isotherm models can be used to effectively model the relationship between moisture content and A_w_ in broiler litter and biochar mixtures.

## Highlights

• Biochar additions to used broiler litter increased the overall water activity of the mixtures.
• Large biochar macropores and hydrophobicity likely led to increased water activity when compared to used broiler litter.
• Moisture sorption isotherms of used broiler litter and biochar mixtures can be characterized using common models.
• Biocha and used broiler litter are characterized by Type III moisture sorption isotherms.

## Graphical abstract

**Figure:**
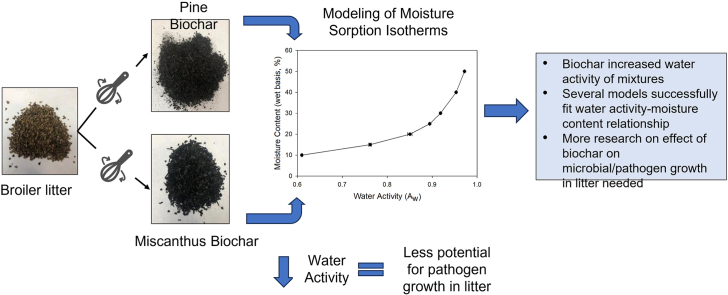

## 1 Introduction

Broiler or meat-type chickens in commercial settings are reared on a variety of absorbent bedding materials including pine shavings, rice hulls, peanut shells, and other wood or grass-based materials. The purpose of the litter is to absorb moisture from bird excreta and water spilled from drinker systems. Factors such as flock-age, litter material, husbandry, ventilation, weather, and season can affect litter moisture. Typical moisture contents are between 15 and 45 % wet basis (wb). Elevated litter moisture can lead to increased ammonia generation that is detrimental to health and bird performance [[1], [2], [3], [4], [5], [6], [7], [8], [9], [10]] and can lead to higher incidences of footpad dermatitis [[11], [12], [13], [14], [15]]. Litter moisture content has also been shown to influence microbial activity, and wet litter provides ideal environments for pathogens and ammonia-generating bacteria to flourish [[16], [17], [18], [19], [20]].

Broiler growers primarily use ventilation to control litter moisture and in-house ammonia concentrations. However, ventilation may be limited to conserve energy, especially during colder months [21]. Growers commonly use acidifying litter amendments to induce short-term reductions of ammonia emissions, but they usually do not affect moisture content in the litter and their efficacy at reducing microbial loads is inconclusive [[22], [23], [24], [25]]. Absorbent litter amendments that improve the water holding capacity of broiler litter may influence microbial communities by limiting water availability; however, their effectiveness has not been examined in the context of live broiler production.

Linhoss et al. [26] explored biochar (BC) as a litter amendment and reported that a 20 % by weight addition of pine BC (PBC) to broiler litter resulted in a 32 % increase in water holding capacity. In a follow up study, Mohammadi-Aragh et al. [27] reported that *Escherichia coli* (*E. coli*) and total aerobic counts decreased with increasing rates of PBC, however, only a 30 % w/w addition was significantly different than the control. They concluded that PBC may be a beneficial litter amendment but did not report water activity (A_w_) values, which is a useful metric to understand a materials susceptibility to microbial growth. A_w_ is described in more detail in the preceding paragraph.

Moisture content of litter materials is an important metric for gauging the potential for microbial and pathogen growth in broiler litter. However, A_w_ is a more descriptive metric that is commonly used in food preservation technology. A_w_, also referred to as equilibrium relative humidity (ERH), is defined as the vapor pressure of water in equilibrium with a material divided by the saturation vapor pressure of water vapor at the same temperature. While moisture content describes how much water is in a material on a weight basis (w/w, %), A_w_ reflects the availability or relative freedom of water (free water) for microbial growth and deteriorative reactions [28]. Past studies have shown that the microbial properties of litter are closely linked to A_w_ and that maintaining A_w_ below thresholds can result in reduced pathogen growth [[16], [17], [29], [30], [31], [32], [33]]. In addition, A_w_ can influence physical handling properties [34]. As mentioned previously, BC has been shown to influence pathogens in various applications; however, there is currently no research available that examines the influence of BC on A_w_ in commercial broiler litter. The research presented here operates under the assumption that BC would be added to broiler litter just prior to flock placement, since this is what is commonly done with other litter amendments.

A_w_ and moisture content are closely related, and their relationship is commonly represented by moisture sorption isotherms. Moisture sorption isotherms are graphs that show the relationship between equilibrium moisture content and A_w_ at a constant temperature and pressure. This relationship depends on the interaction between water and the chemical composition, physical structure, and physical-chemical state of the material. Therefore, moisture sorption isotherms are unique to individual materials.

There are numerous models available to describe moisture sorption isotherms. Most of them use nonlinear regression to represent the relationship between moisture content and A_w_. Bouquet et al. [35] evaluated the usefulness of several two parameter isotherms and found the Halsey and Oswin models to be the most versatile in describing the moisture sorption isotherms of 39 different foods. The Henderson model has also been found to have many practical applications. It is one of the only equations that includes a direct temperature term [36]. Dunlop et al. [37] reported the Henderson model and non-linear (exponential) regression analysis are both useful for modeling broiler litter moisture isotherms. The Guggenheim-Anderson-de Boer (GAB, [28]) model is also widely used, and was considered an improvement over the Brunauer-Emmett-Teller (BET, [38]) model due to application at A_w_ levels greater than 0.5 [28]). Evaluating moisture isotherm models for broiler litter amended with BC can help improve the understanding of how moisture content and A_w_ are related in these heterogenous mixtures.

Materials such as pine shavings and peanut hulls have been used for years as litter in broiler production because they are absorbent. BC also has absorbent properties and has been shown to improve soil water absorption in soil and water holding capacity in broiler litter [26,39,40]. However, BC is generally considered a hydrophobic material [41]. Gray et al. [42] suggested that both the porosity of the feedstock and the production temperature play a role in the water holding capacity of BC. They found that lower production temperatures resulted in greater hydrophobicity and lower overall water uptake. Batista et al. [40] also reports that lower hydrogen and oxygen contents in BC can lead to greater hydrophobicity. The PBC and miscanthus BC (MBC) used in this study were not what is commonly called “engineered biochar,” meaning they were not created under specific conditions to maximize any particular characteristics, such as pore size or hydrophilicity/hydrophobicity. These BCs were chosen because they were readily available in the central Mississippi area. The PBC used was a residual product from the kiln drying of commercial lumber and MBC was being heavily researched as an energy crop at the time and was readily available.

The objectives of this study were to 1) determine the effect of varying inclusion rates of pine and miscanthus BC on A_w_ when mixed with broiler litter, 2) model the relationship between moisture content and A_w_ (moisture sorption isotherms) in broiler litter and BC mixtures, and 3) examine the results in the context of commercial broiler production.

## 2 Materials and methods

### 2.1 Sample collection

PBC was acquired from a lumbermill and produced at temperatures ranging from 700 to 1000 °C. MBC was produced in the Sustainable Bioproducts Department at Mississippi State University. Oven-dried miscanthus was milled to 3 mm before pyrolyzation for 1 min at a temperature of 450 °C following methodologies used by Ingram et al. [43]. At the time the MBC was made, researchers at Mississippi State University were interested in bio-oil production from miscanthus. Research has demonstrated that pyrolysis oils are maximized at temperatures between 425 and 557 °C, and most pyrolysis studies that aim to generate bio-oil use this temperature range [43]. Used pine broiler litter was collected in between flocks from commercial broiler houses at Mississippi State University. Litter from these houses had not been completely removed in over 8+ years at the time of collection. Broiler litter and both BCs used for analysis were sieved to particle sizes ranging from 850 μm to 1400 μm.

### 2.2 Treatments and sample preparation

A 2 × 7 × 7 factorial arrangement of treatments was used with main effects of BC inclusion rate, BC type, and initial moisture content. PBC and MBC were included with broiler litter at the following inclusion rates by weight: 0, 10, 20, 30, 40, 50, and 100 %. A sieve shaker was used to mix combinations of broiler litter and BC for 5 min.

Mixed samples were dried (103 °C) for 24 h and weighed. Distilled water was used to moisten samples to target moisture contents of 10, 15, 20, 25, 30, 40, and 50 % wb. Each sample was covered and left undisturbed for 24 h to stabilize. Wet-basis moisture content was measured on three replicates per inclusion rate of BC (98 total) to compare actual and target moisture contents using the procedure described in ANSI/ASAE S358.3. All measured moisture content values were within 2 % of target. Wet-basis moisture (MC_wb_) content was calculated using Equation (1).

(1) $${MC}_{wb}\left(\%\right)=\frac{Water\;Weight}{Dry\;Sample\;Weight+Water\;Weight}\times 100$$

### 2.3 Morphological characterization

Total pore volume, pore diameter, and specific surface area (SSA) were determined for PBC and MBC using Brunauer-Emmett-Teller (BET) analysis. Analyses were performed using N_2_ adsorption gas at 77K via a porosity and surface area analyzer (Micrometrics, Tristar II Plus, Norcross, GA). Scanning electron microscopy (JEOL, JSM-6500F, Peabody, MA) was used to take images of PBC and MBC.

### 2.4 Moisture sorption isotherms

A soil water potential meter (WP4C, Meter, Pullman, WA) at 25 °C was used to measure A_w_ for each treatment combination. Water potential was converted to A_w_ using Equation (2). Approximately 7 g of a given litter and BC mixture was loaded into WP4C to measure A_w_ using the precise mode (∼10–15 min per sample) at 25 °C. Prior to recording A_w_ data, sample temperatures were checked to ensure they were within 24.5–25 °C per the manufacturer's specifications. Three replicates per treatment combination were measured (n = 294 total). Calibration was performed using a 0.50 mol/kg KCl salt standard. All readings were within an A_w_ of ±0.05 of the KCl standard. Moisture sorption isotherms were generated for all treatment combinations by plotting moisture content as a function of A_w_.

(2) $$A_{w=}e^{\left[\frac{MPa\times 18.02}{8.3143\times \left(T{}^{\circ}C+273.15\right)}\right]}$$

where:

$A_{w}$ = water activity

MPa = megapascals.

T°C = temperature (Celsius)

### 2.5 Moisture sorption isotherm modelling

Halsey, GAB, Oswin, and Henderson models were fitted to the moisture sorption isotherms for all treatment combinations (Table 1). For these models, dry-basis moisture content was plotted as a function of A_w_. Similar to Dunlop et al. [37], an exponential rise to max function was also fitted to the data, except with A_w_ plotted as a function of moisture content. Dry-basis moisture content was used for modelling purposes, but wet-basis moisture content is most commonly used in the poultry industry and therefore, figures and in-text references to moisture content will use wet-basis moisture content. Although the Brunauer-Emmet-Teller (BET) is commonly used in analyses of moisture sorption isotherms, it was not used in this study due to its limited accuracy at water activities below 0.5 [44]. Since data presented in this study and by Dunlop et al. [37] showed that water activities of broiler litter are rarely below 0.5, the BET model was considered inappropriate for this study.

**Table 1:** Moisture sorption isotherm model equations.

Model	Equation	
GAB (van den Berg and Bruin, 1981)	$X=\frac{X_{m}C{K\;A}_{w}}{\left(1-KA_{w}\right)\left(1+\left(C-1\right)A_{w}\right)}$	(3)
Halsey (Halsey, 1948)	$X={\left[\frac{-a}{\ln\left(A_{w}\right)}\right]}^{\frac{1}{b}}$	(4)
Henderson (Henderson, 1952)	$X={\left[\frac{\ln\left(1-A_{w}\right)}{-aT}\right]}^{\frac{1}{b}}$	(5)
Oswin (Oswin, 1946)	$X={a\left[\frac{A_{w}}{1-A_{w}}\right]}^{b}$	(6)
Exponential Rise to Max	$A_{w}=a\left(1-e^{-bX}\right)$	(7)

X = moisture content [dry basis (db)], $A_{w}$ = water activity, a,b , *K,* and *C* are model parameters, T = temperature (K), X_m_ = monolayer moisture content, K = constant related to multilayer sorption heat, C = constant related to monolayer sorption heat.

### 2.6 Statistical methods

PROC NLIN in SAS was used to model all moisture sorption isotherms using equations presented in Table 1. Model fit was determined using the coefficient of determination (R^2^), root mean square error (RMSE), and mean absolute percentage error (MAPE), as defined in equation 3.

(8) $$MAPE\;\left(\%\right)=\frac{100}{n}\sum_{1}^{n}\frac{\left|X_{ei}-X_{pi}\right|}{X_{pi}}$$

where:

MAPE = mean absolute percentage error (%)
$X_{ei}$ = experimental value
$X_{pi}$ = predicted value

## 3 Results and discussion

### 3.1 Biochar A_w_ and physicochemical analysis

There were apparent physicochemical differences between PBC and MBC. Surface area of PBC was roughly 34 times greater than MBC, while the pore volume was nearly 26 times greater (Table 2). The higher surface area and pore volume for PBC most likely resulted from higher pyrolysis temperatures (700–1,000 °C) compared to MBC (450 °C). Water held in larger macropores is generally bound less tightly than in micropores [45], which could have led to the slightly higher A_w_ for PBC when compared to MBC (0.87 vs 0.85, respectively).

**Table 2:** Physicochemical properties of pine biochar, miscanthus biochar, and broiler litter.

Parameter	Pine Biochar (PBC)	Miscanthus Biochar (MBC)	Broiler litter
N_2_ BET Specific Surface Area (m^2^/g)	34.65	1.14	2.55
Pore Volume (cm^3^/g)	0.00947	0.00043	0.00038
Pore Diameter (Å)	10.93	11.76	5.93
Carbon (C)	97.25	69.07	33.97
Hydrogen (H)	0.70	3.54	4.96
Oxygen (O)	1.59	26.80	24.13
Nitrogen (N)	0.41	0.50	3.80
Sulfur (S)	0.046	0.085	1.08
Ash (g)	0.008	0.002	0.04

A meta-analysis of feedstock on BC characteristics reported elevated carbon content in BC derived from woody materials as compared to grasses [46]. In this study, carbon content of PBC (woody material) was 97.3 %, while only 69.1 % for MBC (grass material). Higher pyrolysis temperatures have also been shown to result in reduced hydrogen and oxygen levels [46]. Table 2 shows PBC had lower hydrogen (0.7 %) and oxygen (1.6 %) composition compared to MBC (hydrogen = 3.5 %, oxygen = 26.8 %). Lower hydrogen and oxygen contents in BC have been shown to increase hydrophobicity [40]. It is possible that the lower hydrogen and oxygen levels in the PBC lead to an increased hydrophobicity, thereby increasing A_w_ when compared to MBC.

**Fig. 1:**
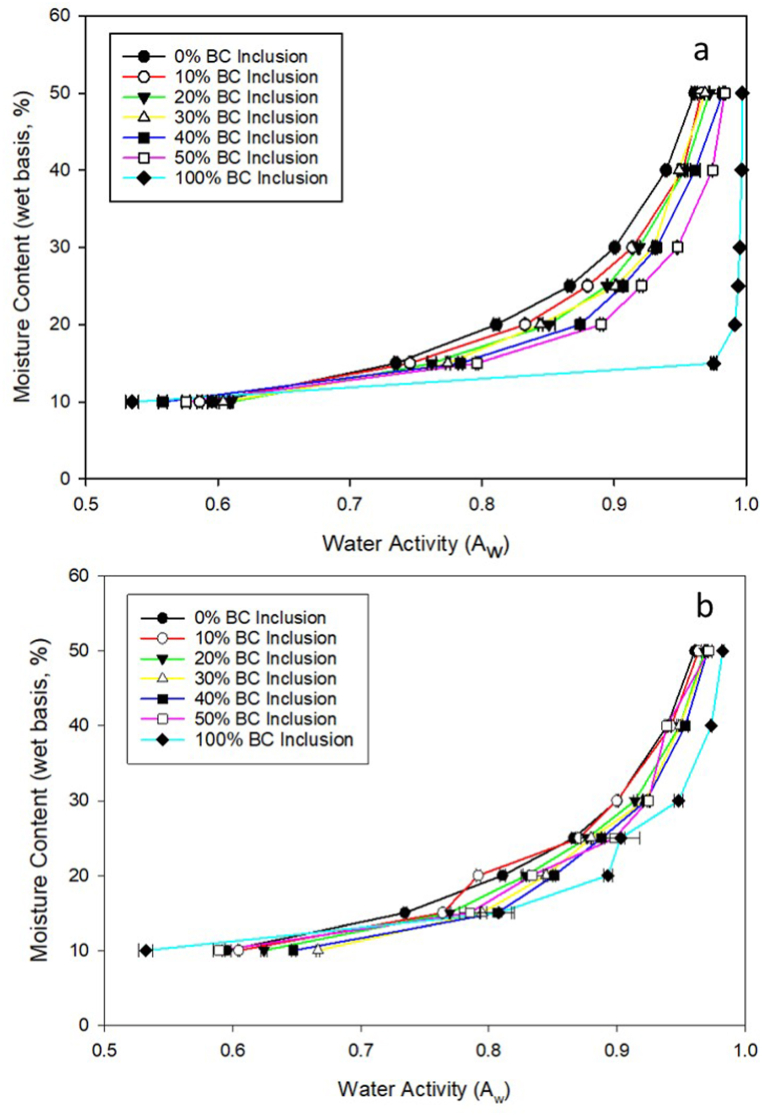
Moisture sorption isotherms for broiler litter mixed with varying inclusion rates of PBC (a) and MBC (b).

Fig. 1a and b shows A_w_ and moisture content at moisture contents most common in commercial broiler houses. Moisture contents of 40 and 50 % wb in commercial houses would mostly likely lead to bird health concerns but were included here for modeling purposes. Moisture content increased exponentially for both PBC and MBC as A_w_ increased (Fig. 1a and b). For the same moisture content, A_w_ generally increased with increasing rates of BC inclusion, which suggests that BC inclusion in broiler litter increases its A_w_.

**Fig. 2:**
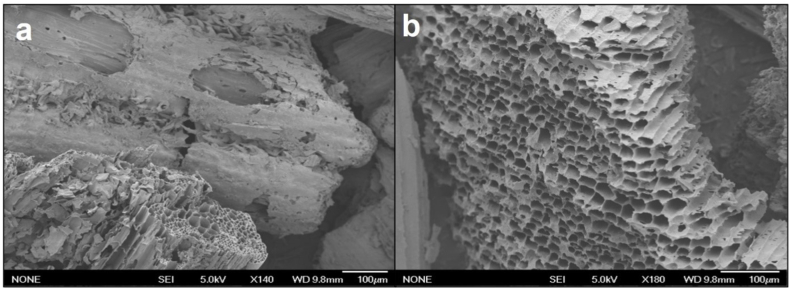
Scanning electron microscopy (SEM) images of MBC (a; × 140 magnification) and PBC (b; × 180 magnification).

A_w_ for 100 % PBC increased from 0.54 to 0.98 when moisture content increased from only 10–15 % wb. The authors hypothesize the increase in moisture content from 10 to 15 % wb provided enough water to fully saturate the micropores (<0.2 μm) of PBC and begin to fill the larger macropores (>10 μm). The elevated surface area of PBC indicates an abundance of micropores [47], which have been shown to bind water more tightly than macropores [45]. Therefore, the low A_w_ at 10 % wb moisture content for PBC may be a result of the water being held more tightly within the micropores. However, the water that began to fill the macropores at around 15 % wb moisture content may not have been bound tightly, leading to a high A_w_. Scanning electron microscopy (SEM) shows the visual representations of the porous structures of PBC and MBC (Fig. 2a and b).

### 3.2 Modeling results

Table 3, Table 4 show model results for the GAB, Halsey, Henderson, and Oswin models at each BC inclusion rate for pine and miscanthus, respectively. Higher R^2^ and lower RSME and MAPE represent better fits for each model. Considering only R^2^ values and RSME, all the models seem to perform well. However, MAPE values of <10 % are considered indicative of a good fit for practical purposes. For the PBC, Halsey and Oswin models are the only ones that have an average MAPE values of <10 % (8.50 and 8.45, respectively) (Table 3). For MBC, MAPE values for the GAB, Halsey and Oswin models are below 10 % (8.35, 4.87, & 5.80, respectively) (Table 4). In general, goodness of fit decreased for both PBC and MBC at the highest inclusion rates of BC (50 and 100 %).

**Table 3:** Parameters for moisture sorption isotherms, R^2^, RMSE, and mean absolute percentage error (MAPE) for used broiler litter with different PBC inclusion rates at 25 °C.

PBC Inclusion Rate	Model	R2	RMSE	MAPE (%)	a	b	K	Xm	C
0	GAB	0.9991	0.0085	4.18			0.9942	0.044	4790.2
10	GAB	0.9874	0.0325	20.21			0.9994	0.0313	76.1
20	GAB	0.9990	0.0325	9.61			0.9912	0.0355	169.8
30	GAB	0.9916	0.0266	15.82			1.0012	0.0293	889.4
40	GAB	0.9945	0.0215	10.52			0.9823	0.0343	115.0
50	GAB	0.9801	0.0409	27.59			0.9959	0.0195	2219.5
100	GAB	0.9554	0.0613	35.20			0.9862	0.00776	875.9
**AVG**		0.9867	0.0320	17.58					
0	Halsey	0.9995	0.0065	1.55	0.0398	1.1648			
10	Halsey	0.9922	0.0257	5.72	0.0332	1.1982			
20	Halsey	0.9996	0.0057	2.07	0.0284	1.2742			
30	Halsey	0.9915	0.0267	8.65	0.0313	1.146			
40	Halsey	0.9927	0.0248	5.15	0.0187	1.5461			
50	Halsey	0.9919	0.0262	7.02	0.0164	1.431			
100	Halsey	0.9417	0.0700	29.38	0.0030	0.7253			
**AVG**		0.9870	0.0265	8.50					
0	Henderson	0.9859	0.0344	13.45	0.0110	0.4636			
10	Henderson	0.9709	0.0494	16.49	0.0117	0.4593			
20	Henderson	0.9870	0.0331	13.82	0.0121	0.4591			
30	Henderson	0.9707	0.0496	19.97	0.0118	0.4118			
40	Henderson	0.9882	0.0315	13.95	0.0135	0.5063			
50	Henderson	0.9663	0.0532	18.61	0.0141	0.4583			
100	Henderson	0.9296	0.0769	32.22	0.0195	0.1317			
**AVG**		0.9712	0.0469	18.35					
0	Oswin	0.9987	0.0103	3.60	0.0733	0.8146			
10	Oswin	0.9903	0.0286	7.43	0.0672	0.7954			
20	Oswin	0.9991	0.0087	3.70	0.0689	0.7534			
30	Oswin	0.9896	0.0295	0.03	0.0554	0.838			
40	Oswin	0.9932	0.0238	6.26	0.0832	0.626			
50	Oswin	0.9899	0.0291	8.72	0.0616	0.678			
100	Oswin	0.9413	0.0702	29.41	0.0003	1.3766			
**AVG**		0.9860	0.0286	8.45

**Table 4:** Parameters for moisture sorption isotherms, R2, RMSE, and mean absolute percentage error (MAPE) for used broiler litter with different MBC inclusion rates at 25 °C.

MBC Inclusion Rate	Model	R2	RMSE	MAPE (%)	a	b	K	X_m_	C
0	GAB	0.9992	0.0084	3.32			0.9941	0.0445	4417.2
10	GAB	0.9969	0.0162	4.29			0.9899	0.0455	450.9
20	GAB	0.9983	0.0120	4.53			0.9907	0.0401	322.9
30	GAB	0.9972	0.0155	4.43			0.9892	0.0404	13.6
40	GAB	0.9982	0.0122	4.45			0.9928	0.0357	1777.1
50	GAB	0.9768	0.0442	9.20			0.9844	0.0442	7.2051
100	GAB	0.9728	0.0478	28.28			0.9948	0.0204	208.6
**AVG**		**0.9913**	**0.0223**	8.35					
0	Halsey	0.9995	0.0065	1.55	0.0398	1.1648			
10	Halsey	0.9975	0.0144	4.72	0.0368	1.2212			
20	Halsey	0.9993	0.0078	2.58	0.0321	1.2404			
30	Halsey	0.9968	0.0165	4.63	0.0305	1.2214			
40	Halsey	0.9988	0.0101	3.22	0.0296	1.2057			
50	Halsey	0.9725	0.0480	7.90	0.0295	1.3043			
100	Halsey	0.9860	0.0343	9.50	0.0167	1.4467			
**AVG**		**0.9929**	**0.0197**	4.87					
0	Henderson	0.9859	0.0344	13.45	0.0110	0.4636			
10	Henderson	0.9870	0.3310	11.54	0.0113	0.4804			
20	Henderson	0.9884	0.3128	11.52	0.0118	0.4675			
30	Henderson	0.9911	0.0273	9.62	0.0119	0.4479			
40	Henderson	0.9892	0.0301	10.95	0.0120	0.4359			
50	Henderson	0.9701	0.0502	16.38	0.0120	0.4653			
100	Henderson	0.9600	0.0580	20.91	0.0140	0.4696			
**AVG**		**0.9817**	**0.1205**	**13.48**					
0	Oswin	0.9987	0.0103	3.60	0.0733	0.8146			
10	Oswin	0.9974	0.0149	4.30	0.0774	0.7783			
20	Oswin	0.9990	0.0089	2.77	0.0712	0.7705			
30	Oswin	0.9972	0.0154	5.05	0.0655	0.7838			
40	Oswin	0.9986	0.0110	4.32	0.0609	0.7969			
50	Oswin	0.9733	0.0474	9.24	0.0753	0.7366			
100	Oswin	0.9838	0.0369	11.33	0.0646	0.6699			
**AVG**		**0.9926**	**0.0207**	**5.80**					

**Fig. 3:**
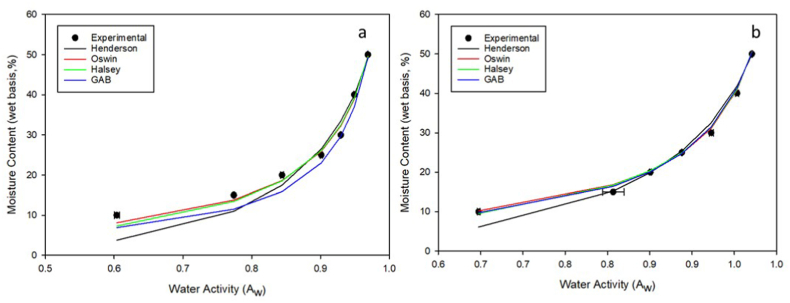
Experimental and predicted moisture sorption isotherms of broiler litter with a 30 % PBC (a) and MBC (b) inclusion rate at 25 °C.

Fig. 3a and b shows experimental and predicted results for the GAB, Halsey, Henderson, and Oswin models at a 30 % PBC and MBC inclusion rate. Only data at 30 % BC inclusion rate is presented here for brevity, and because previous studies [26,27] reported that 20–30 % BC w/w inclusion rates may be most applicable for broiler production from an ease of application and water holding capacity standpoint. At 30 % wb moisture content, BC can absorb 3.5 times its weight in moisture. However, due to its low bulk density, inclusion rates above 30 % become unmanageable as a litter amendment due to the volume of material that must be applied to the litter.

Similar to the moisture isotherms presented by Bernhart and Fasina [34], those presented in Fig. 3a and b are type III isotherms that are typically obtained from materials with elevated amounts of soluble components such as sugar and salts. Bernhart and Fasina [34] reported that broiler litter may contain up to 40 % sugars (hemicellulose and cellulose), which mostly likely explains the type III isotherms presented here.

**Fig. 4:**
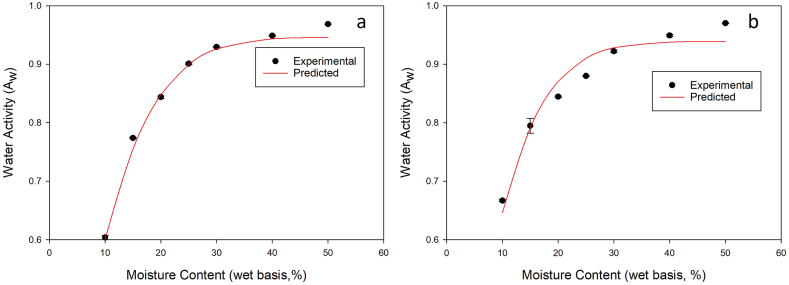
Experimental and predicted moisture sorption isotherms of broiler litter with a 30 % PBC (a) and MBC (b) inclusion rate at 25 °C. Isotherms were fitted using an exponential rise to max function.

Most of the food storage and preservation literature that models moisture sorption isotherms does so with moisture content plotted as a function of A_w_. However, Dunlop et al. [37] used an exponential rise to max function to model A_w_ as a function of moisture content for broiler litter sampled at different times during a growout and reported R^2^ values as high as 0.983. In this study, the exponential rise to max function accurately predicted A_w_ except at the 100 % PBC inclusion rate (Table 5). MAPE was lower for the exponential rise to max model than for any of the other models, primarily because it did a better job of predicting values at higher BC inclusion rates. Fig. 4a and b shows plots of experimental and predicted A_w_ values for PBC and MBC at a 30 % inclusion rate for the exponential rise to max model.

**Table 5:** Exponential Rise to Max model parameters, R2, RMSE, and mean absolute percentage error (MAPE) for used broiler litter with different BC inclusion rates at 25 °C.

	BC Inclusion Rate	T (°C)	R2	RMSE	MAPE (%)	a	b
Pine	0	25	0.9826	0.0150	1.76	0.9368	8.5979
	10	25	0.9935	0.0101	1.08	0.9509	8.4756
	20	25	0.9923	0.0104	0.98	0.9534	9.0106
	30	25	0.9941	0.0091	0.90	0.9548	9.0310
	40	25	0.9780	0.0200	2.31	0.9729	8.4267
	50	25	0.9816	0.0181	1.93	0.9815	8.6888
	100	25	0.7585	0.0785	7.25	1.026	9.7341
	AVG		0.9544	0.0231	2.31		
Miscanthus	0	25	0.9826	0.0157	1.756	0.9368	8.5979
	10	25	0.9569	0.0239	2.587	0.9390	8.9178
	20	25	0.9739	0.0179	1.985	0.9422	9.333
	30	25	0.9517	0.0213	2.163	0.9387	10.4945
	40	25	0.9517	0.0213	1.791	0.9387	10.4945
	50	25	0.9821	0.0162	1.738	0.9514	8.9723
	100	25	0.9473	0.0334	3.490	0.9839	8.2901
	**AVG**		0.9637	**0.0214**	2.21

**Fig. 5:**
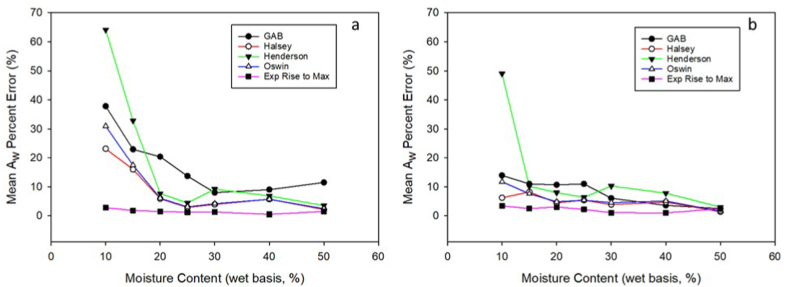
Percent error for all models at different moisture contents for both PBC (a) and MBC (b).

Fig. 5a and b shows that the models seemed to perform worse at lower moisture contents, especially for PBC. For PBC, none of the models except the exponential rise to max function had mean A_w_ percent error of less than 10 % for moisture contents ranging from 10 to 15 % wb. There was less variation in mean A_w_ percent error for PBC (excluding Henderson) at lower moisture contents. Across all moisture contents for MBC except 50 % wb, the exponential rise to max function exhibited the lowest mean A_w_ percent error. In addition, there was less variation in mean A_w_ percent error for all models (except GAB for PBC) at the highest moisture contents (40–50 % wb), which indicates they all predict better at higher moisture contents.

Of the models tested, the exponential rise to max function seems to be the most useful, primarily because it is most accurate in moisture contents that are typically found in broiler house environments 15–45 % wb). In addition, broiler industry representatives are more accustomed to discussing litter in terms of moisture content than A_w_, so plotting A_w_ as a function of moisture content is more intuitive. However, the Oswin and Halsey models are also suitable models, as they are more accurate at moisture contents within common poultry house ranges.

Bernhart and Fasina [34] reported an A_w_ of 0.90 for broiler litter at moisture contents of 27 % wb. Microorganisms found in broiler houses, such as *E. coli, Salmonella* spp., *Staphylococcus* spp., *Aspergillus* spp., and *Clostridium* spp., thrive in A_w_ between 0.75 and 0.98 [[48], [49], [50], [51]]. However, A_w_ of less than roughly 0.90 are generally recommended in broiler litter to prevent mold and bacteria growth [37]. Experimental results presented in Fig. 4 show that broiler litter amended with 30 % PBC and MBC had an A_w_ of 0.90 at 25 % and 27 % moisture content wb, respectively. However, raw broiler litter (0 % BC inclusion rate) was found to have an A_w_ of 0.90 at 30 % moisture content wb. As mentioned previously, both PBC and MBC were found to increase A_w_ when mixed with broiler litter, thereby leading to a higher A_w_ at the same moisture content. Dunlop et al. [37] reported that for the same moisture content, A_w_ decreased over the course of a commercial flock, even with the continual addition of manure. They attributed this to the activity of the birds (walking, scratching, foraging), which can continually coat excreta in drier litter and draw water out it. Ventilation is also a critical aspect of commercial production that is used to dry litter and reduce A_w_. It is important to note that for this study, distilled water was used to alter moisture contents in the broiler litter – BC mixtures. It is currently unclear how additions of BC to litter would affect A_w_ in a commercial setting; therefore, further investigation is warranted.

Although addition of BC to broiler litter seems to increase A_w,_ Mohammadi-Aragh et al. [27] did not report an increase microbial growth in litter amended with up to 30 % BC w/w when compared to a control with no BC added. Therefore, more research is needed to determine the influence of BC addition to broiler litter on microbial growth.

Broiler chickens are continually in contact with the litter material on which they are raised. Therefore, litter management to reduce ammonia and potential pathogens is critical for the performance and welfare of the birds. It is well understood that A_w_ is an indicator of litter properties and is linked to microbial activity. Therefore, prior to serious investigation of BC as a potential broiler litter amendment, an understanding of its potential effects on A_w_ and microbial growth are warranted. While A_w_ may change based on litter material and litter characteristics (i.e., age, management strategies, bird stocking density), results from this paper show that several moisture isotherm models can be used to effectively model the relationship between moisture content and A_w_ in broiler litter and BC mixtures.

## 4 Conclusions

PBC had a higher pore volume and surface area than miscanthus, likely resulting from a higher pyrolysis temperature (700–1000 °C vs 450 °C). Although both PBC and MBC additions to used broiler litter increased the overall A_w_ of the mixtures, additional studies are needed that examine their role in altering the microbial communities. The exponential rise to max function most accurately predicted the relationship between moisture content and A_w_ at moisture contents commonly found in broiler houses. In addition, the Oswin and Halsey models performed better than the GAB and Henderson models.

## Data availability

Data will be made available upon request.

## CRediT authorship contribution statement

**John Emerson Linhoss:** Writing – review & editing, Writing – original draft, Methodology, Formal analysis, Conceptualization. **Maryam Kate Mohammadi-Aragh:** Writing – review & editing, Data curation. **Jeffrey Dwayne Evans:** Methodology.

## Declaration of competing interest

The authors declare the following financial interests/personal relationships which may be considered as potential competing interests:John Linhoss reports financial support was provided by 10.13039/100000199USDA.
